# The Effects of Platelet-Rich Fibrin in the Behavior of Mineralizing Cells Related to Bone Tissue Regeneration—A Scoping Review of In Vitro Evidence

**DOI:** 10.3390/jfb14100503

**Published:** 2023-10-09

**Authors:** Renata de Lima Barbosa, Emanuelle Stellet Lourenço, Julya Vittoria de Azevedo dos Santos, Neilane Rodrigues Santiago Rocha, Carlos Fernando Mourão, Gutemberg Gomes Alves

**Affiliations:** 1 Clinical Research Unit, Antonio Pedro Hospital, Fluminense Federal University, Niteroi 24033-900, Brazil; 2 Graduate Program in Science and Biotechnology, Fluminense Federal University, Niteroi 24210-201, Brazil; 3 Department of Periodontology, Tufts University School of Dental Medicine, Boston, MA 02111, USA

**Keywords:** review, osteoblast, cell therapy, PRF, platelet concentrates

## Abstract

Platelet-rich fibrin (PRF) is a second-generation blood concentrate that serves as an autologous approach for both soft and hard tissue regeneration. It provides a scaffold for cell interaction and promotes the local release of growth factors. PRF has been investigated as an alternative to bone tissue therapy, with the potential to expedite wound healing and bone regeneration, though the mechanisms involved are not yet fully understood. This review aims to explore the in vitro evidence of PRF’s effects on the behavior of mineralizing cells related to bone tissue regeneration. A systematic electronic search was conducted up to August 2023, utilizing three databases: PubMed, Web of Science, and Scopus. A total of 76 studies were selected, which presented in vitro evidence of PRF’s usefulness, either alone or in conjunction with other biomaterials, for bone tissue treatment. PRF membranes’ influence on the proliferation, differentiation, and mineralization of bone cells is linked to the constant release of growth factors, resulting in changes in crucial markers of bone cell metabolism and behavior. This further reinforces their therapeutic potential in wound healing and bone regeneration. While there are some notable differences among the studies, the overall results suggest a positive effect of PRF on cell proliferation, differentiation, mineralization, and a reduction in inflammation. This points to its therapeutic potential in the field of regenerative medicine. Collectively, these findings may help enhance our understanding of how PRF impacts basic physiological processes in bone and mineralized tissue.

## 1. Introduction

In recent years, there has been significant advancement in bone tissue engineering, where damaged or diseased bones are repaired using materials that closely replicate the properties of natural bones while being safe for the body. A wide range of both synthetic and natural biomaterials have been extensively studied, each with its unique strengths and limitations. Being aware of the differences between these materials is vital for harnessing their respective benefits and pushing the boundaries of this field [1,2,3].

Various artificially crafted (alloplastic) materials can be customized to meet specific medical requirements nowadays. These synthetic biomaterials made from metals, ceramics, and polymers can be manufactured reproducibly on a large scale, with tunable physical and chemical properties. Nevertheless, these materials usually lack the intricate structure and biological components found in natural materials of biological origin, such as xenografts, allografts, and autografts [1,3]. As a result, these materials of natural origin often have some advantages over synthetic biomaterials, such as increased biocompatibility, biodegradability, and biological activity.

In this context, the development of autologous blood-derived aggregates, such as platelet-rich fibrin (PRF) or leukocyte- and platelet-rich fibrin (L-PRF), has garnered significant interest in the medical and dental fields for its importance in tissue regeneration [2]. PRF stands out as a promising choice for natural biomaterial due to its remarkable compatibility with the human body. As a second-generation blood concentrate, PRF has undergone significant advancements since its inception. It has built on the foundations of platelet-rich plasma (PRP) from the late 1990s and boasts enhanced attributes that make it valuable in numerous surgical procedures [4,5,6,7]. Its concentration of essential cytokines and growth factors facilitates cell growth, migration, proliferation, and differentiation, allowing for smooth integration with native tissues [1,2,3]. 

**Figure 1:**
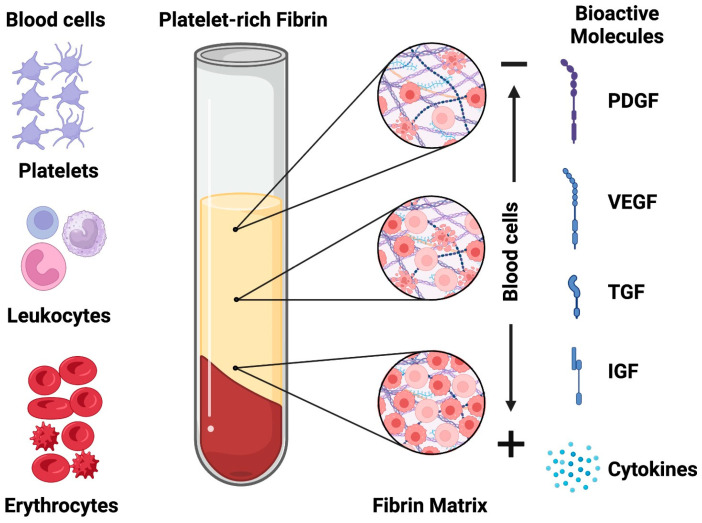
The platelet-rich fibrin membranes consist of cells, structural proteins, and regulatory mediators, such as cytokines and growth factors.

Produced through the straightforward centrifugation of a patient’s blood without the need for anticoagulants or activating agents, PRF manifests as a dense fibrin matrix rich in leukocytes, platelets, and a myriad of proteins pivotal for wound healing. This autologous biomaterial has been hailed for fostering angiogenesis, promoting cell specialization and differentiation, and serving as a supportive scaffold for bone cells, thereby expediting the bone healing and formation process [3]. Furthermore, the polymerization of PRF results in a membrane with a favorable physiological structure, offering a robust framework for osteoprogenitor cells to adhere to and facilitating the continuous release of vital growth factors such as fibroblast growth factors (FGFs), vascular endothelial growth factors (VEGFs), transforming growth factors (TGFs), and platelet-derived growth factors (PDGFs) [8,9,10] (Figure 1). These are important cytokines that regulate various aspects of bone regeneration. While the VEGF stimulates angiogenesis and osteogenesis, the PDGF promotes chemotaxis and the proliferation of mesenchymal stem cells and osteoblasts, the FGF enhances osteoblast differentiation and bone matrix formation, and the TGF modulates inflammation and immune response. These cytokines act synergistically to enhance bone healing and repair, and their delivery to bone defects can improve the outcome of bone regeneration therapies [11,12,13,14].

Despite the promising attributes of PRF, the scientific community continues to explore and debate its clinical efficacy compared to other biomaterials or conventional techniques. Particularly, inquiries concerning its direct positive influence on mineralizing cells, such as osteoblasts, have spurred numerous in vitro studies aimed at unraveling the biological mechanisms and impacts of PRF on mineralized tissues [8,10]. This scoping review aimed to elucidate the understanding of the biological basis of the applicability of different PRF derivations with potential use in bone therapy, compiling and comparing their effects on events such as proliferation, differentiation, and mineralization capacity of bone cells from evidence provided by controlled in vitro studies.

## 2. Materials and Methods

### 2.1. Protocol and Registry

This scoping review was conducted based on the Preferred Reporting Items for Systematic Reviews and Meta-Analysis (PRISMA) Statement and its extension for Scoping Reviews (PRISMA-ScR) [15]. The present research protocol is registered in the Open Science Framework database. 

### 2.2. Information Sources and Search Strategy

The search strategy was developed based on a PICOS framework, where the following aspects were considered. Population: cell lines involved in bone regeneration; intervention: exposure to second-generation autologous platelet aggregates (PRFs); comparison: cells not exposed to PRFs; outcome: positive effect on parameters related to regeneration (proliferation, differentiation, mineralization); and setting: in vitro assessments.

The search was conducted up to August 2023 in three different electronic databases: PubMed, Web of Science, and Scopus. The search key employed for each database is described in Table 1. Adaptations were performed to fit the same terms into the different search engines and in combination with specific database filters when available. The entries were sent to Mendeley Desktop (Elsevier) software (version 2.98.0) to eliminate replicas and thus consolidate the list of references for subsequent analysis.

### 2.3. Study Selection

The eligibility criteria for the studies included the use of cell lines for bone regeneration, second-generation autologous platelet aggregate (PRF) exposure to cells, and a positive effect on parameters related to regeneration in vitro. Exclusion criteria comprised study designs and publication types that used other autologous materials that did not involve cell exposure or were case reports, exposure limited to non-mineralizing cells, such as fibroblasts and osteoclasts, articles that did not represent complete primary sources of evidence (abstracts, reviews, editorial letters, opinion letters, commentary articles), and in vivo or clinical studies. Subjects related to other research topics, such as meniscus, cartilage, cancer treatments, drug studies, and in vitro delivery, were considered “off-topic”. There was no restriction on the publication date or language of publication.

Two reviewers (R.L.B. and E.S.L.) read all the titles and abstracts of the articles retrieved from the search after performing piloting and calibrating on data collection with a Cohen kappa of 0.97 concordances. They analyzed and selected articles according to the eligibility criteria described above, eliminating duplicates. Any disagreement on study eligibility was resolved through discussion and consensus or a third reviewer (G.G.A.) to make the final decision.

### 2.4. Critical Appraisal

The selected studies were evaluated by 3 reviewers (R.L.B., N.R.S.R., and G.G.A.) using the ToxRTool (Toxicological Data Reliability Assessment Tool) to assess the quality and reliability of in vitro data at the methodological level [16]. This tool includes 18 criteria that describe important aspects for developing reliable articles regarding the methodology applied and how the study was conducted. The checklist includes a description of the methodological aspects of each study, such as substance identification, test system, study design, and documentation of results. Each criterion corresponds to one point, where the article receives one point if it meets the criterion and zero points if it does not. Finally, the scores are summed, generating a final score. Articles with scores less than 11 are considered unreliable, studies with scores between 11 and 14 are considered reliable with possible restrictions, and studies with scores between 15 and 18 are considered reliable without restrictions.

### 2.5. Data Extraction

For data extraction, scientific and technical information were tabulated and analyzed using Microsoft Office Excel 2013. The data extracted included the authors and year of publication, cell type, growth factors detected, the presence of association/modification/addition with PRF membranes, conditions of exposure of cells to PRF (time, volume), biological parameters evaluated, and main findings for the outcomes. These data were used for a qualitative evaluation and synthesis of evidence.

## 3. Results

**Figure 2:**
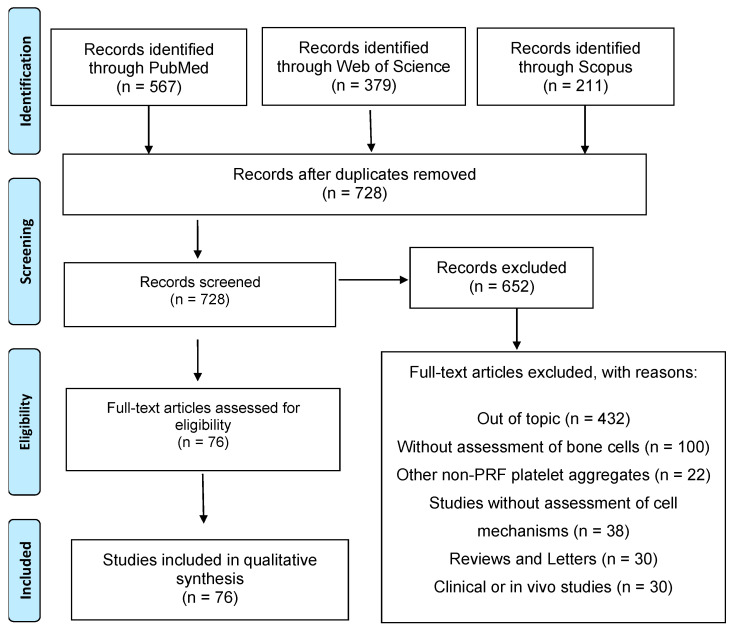
The screening process, selection, and systematic steps according to the PRISMA Statement.

From the search key developed according to each database, 1157 articles were retrieved, and 429 duplicate records were excluded. Thus, 728 articles were evaluated for eligibility, applying the inclusion and exclusion criteria. Of these, 652 articles were excluded from the review because they did not meet the eligibility criteria (Figure 2). Therefore, 76 articles were evaluated in detail and used in the qualitative analysis of this review.

The ToxRTool was employed to assess the inherent quality of the selected studies of toxicological data reported in a publication or a test report, with a reliability categorization performed as shown in Table 2. Among the 76 selected articles, around half (52%) achieved the maximum score (18 points), while a further 48% (36 studies) presented only a few reporting limitations, with scores ranging from 15 to 17, rending a total of 100% of the studies classified as reliable without restrictions (Table 2).

Of these, many articles did not specify the amount of medium used for membrane cultivation/extraction of PRF membranes, the number of replicates, or the passage of cells used in the experiments. These factors can influence the interpretation of the biological response, as the time of cultivation and sequential passages can affect the behavior of cells, changing their morphological, functional, and molecular characteristics, which may compromise the quality and reliability of the results obtained with in vitro cultured cells. 

Table 3 shows the main data extracted from the selected studies regarding the effects of different protocols of PRF production on bone-related cells. Various studies propose ways to obtain and use PRF to optimize its handling characteristics and storage and develop new application possibilities. These include the tube type, time and speed of centrifugation, mode of application, and even association with other materials. 

More than half (56%) of the studies investigated the classical L-PRF protocol proposed by Choukx, which forms a thick clotted yellow, Jell-O-like moldable scaffold. Advanced PRF (A-PRF) is another type of platelet concentrate produced by centrifuging blood at a low speed and for a longer time, which results in a fibrin clot with a rich content of platelets, growth factors, and other bioactive molecules, which was studied in nine (12%) of the selected studies. Further, nine studies investigated the effects of exposure to injectable PRF (i-PRF), which is usually prepared by centrifuging blood samples at 1060 rpm for 14 min, forming a thinner unclotted liquid that can be injected into various sites where bone grafts have been placed or where infections have occurred. Two other studies assessed the biological properties of H-PRF, which is produced by horizontal centrifugation. Finally, nine studies (12%) investigated the impact of freezing or lyophilizing L-PRF membranes on the effects of these materials on mineralizing cells. A few of the selected studies (six) compared the biological properties of two or more of these protocols, as will be discussed in the next section of this review. 

One of the challenges in studying the effects of PRF on cell behavior is to choose the appropriate method of exposure. About half (51%) of the selected studies employed indirect exposure of cells to eluates, while the other 42% (32 studies) cultivated cells in co-culture or directly seeded into PRF matrices. The difference between direct and indirect in vitro exposure of cells to PRF eluates or co-cultures is that the former simulates the initial contact of cells with PRF, while the latter mimics the long-term interaction of cells with PRF. The direct exposure of cells to PRF eluates can assess the cytotoxicity, inflammation, and differentiation potential of PRF, while the co-culture of cells with PRF can evaluate the proliferation, migration, and mineralization ability of cells in the presence of PRF.

The name of a protein indicates the assessment of its expression. L-PRF: fibrin rich in platelets and leukocytes produced by Choukrun’s protocol; i-PRF: liquid, injectable PRF; A-PRF: advanced PRF, produced with intermediary centrifugation forces; H-PRF: produced through horizontal centrifugation. RCF: relative centrifugal force; ALP: alkaline phosphatase; OPN: osteopontin; OPG: osteoprotegerin; DMP-1: dentin matrix protein 1; DSPP: dentin sialophosphoprotein; DSP: dentin sialoprotein; SPP: sialophosphoprotein; BSP: bone sialoprotein; OCN: osteocalcin; Col-1: collagen type I; OSX: osterix; MMP: matrix metalloproteinase; VCAM-1: vascular cell adhesion molecule 1; BMP-2: bone morphogenetic protein 2; MGP: matrix Gla protein; LOX: lysyl oxidase; LPS: lipopolysaccharide; P-GSK3b: phosphorylated glycogen synthase kinase-3-beta.

Regarding the cell types employed in the studies, several different models were identified from diverse mineralizing tissues of both human and animal origin. More than half of the studies (n = 44, 58%) employed human cells, while the other half investigated cells from animal sources (42%), including cells from rabbits, canines, and murine models. None of the selected studies performed a comparison between human or animal cells in order to provide direct evidence of different responses to PRF between these origins. All studies that performed assessments of cell viability and proliferation after exposure of either human or animal cells to PRF presented similar descriptive results, even with incomparable size effects, as the methodological settings were very heterogeneous (the tables include records of studies without any data). Furthermore, very similar regulatory pathways are reported as activated by exposure to PRF, including the Cbfa-1/Runx2, MAPK, and Wnt signaling pathways, regardless of the human or animal origin of cells, as will be further described and discussed in the next section of this review. Nevertheless, it is important to notice that data resulting from human cells are usually considered more relevant and representative of human physiology and pathology than animal cells, which may have different molecular and cellular mechanisms, responses, and interactions. Furthermore, their obtention may reduce ethical and practical issues associated with the use of animals for research, such as animal welfare, availability, cost, and regulatory approval.

Several of the selected studies utilized primary cells, such as osteoblasts, dental pulp stem cells (hDPSCs), periodontal ligament cells (cPDLs), and bone marrow stem cells (BMSCs) (Table 4). Primary cells are isolated directly from living tissues or organs and offer several advantages over immortalized cell lines. They retain the physiological functions of their tissue of origin, such as gene expression, metabolism, and responsiveness to stimuli. They provide a more realistic model system, making them more suitable for studying complex biological processes. However, immortalized cell lines, which have been modified to proliferate indefinitely in culture, offer some benefits over primary cells. They are more homogeneous and consistent, easier to maintain and manipulate, and more readily available and cost-effective. Some of the selected studies utilized immortalized cell lines, including the well-established preosteoblasts from rat calvaria MC3T3-E1 and the human osteosarcoma cell lines Saos-2, MG-63, and U2OS (Table 4).

The majority of the selected articles investigated cells in monoculture. A single exception is the study by Dohle et al. [29], which employed a co-culture of outgrowth endothelial cells (OECs) and primary osteoblasts (pOBs) exposed together to injectable PRF. The authors presented evidence that i-PRF may have a positive effect on wound healing processes and the angiogenic activation of endothelial cells, as the expression of E-selectin, ICAM-1, VEGF, and ALP was significantly higher in the exposed cells. OECs are a subpopulation of endothelial progenitor cells (EPCs) that have high proliferative and angiogenic potential, which have been already shown to interact with osteoblasts in a positive manner, enhancing osteogenic differentiation and increased mineralization. Additionally, osteoblasts secrete IL-8, which enhances the migration, survival, and expression of angiogenic factors and matrix metalloproteinases [92]. In this context, identifying that PRF may enhance these interactions may provide an interesting tool for bone tissue engineering.

Five studies investigated more than one cell type with the same methodology, even though not in co-culture, providing data that allowed us to identify some cell type-specific differences in the response to PRF preparations. The study by Dohan Ehrenfest et al. [28] was one of the first identified assessing different responses to L-PRF by exposing gingival fibroblasts, dermal prekeratinocytes, preadipocytes, and maxillofacial osteoblasts. The results showed that PRF continually stimulates proliferation in all studied cell types, but this stimulation was stronger and dose-dependent in osteoblasts, while it was observed only on day 14 with fibroblasts. Adipocytes and prekeratinocytes also differed by presenting increased metabolic activity (as detected by mitochondrial activity) and were probably related to different regulations of metabolism in these cells. Clipet et al. [27] also compared human osteoblasts (Saos-2), fibroblasts (MRC5), and epithelial (KB) cell lines. Similar to the findings of Dohan et al., while PRF increased the proliferation of all cell types, the effects were more evident in osteoblasts at shorter experimental times. Gassling et al. [35], 2009, also compared the exposure of human osteoblasts, human fibroblasts, and human osteoblast-derived osteosarcoma cells (Saos-2) to PRF, assessing their effects not on proliferation but on the induction of the release of growth factors by these cells. While growth factors could be detected in all of the samples, fibroblasts secreted lower levels of PDGF-AB, PDGF-BB, IGF-I, and TGF-ß1 than osteoblasts, especially those derived from osteossarcoma, suggesting increased paracrine activation by PRF in transformed cells. A similar pattern was also observed in the study by Kardos et al. [47], 2018, where mesenchymal stem cells presented higher rates of proliferation when exposed to fresh or lyophilized PRF samples compared to gingival fibroblasts. However, a recent study by Al-Maawi et al. [18], 2022, comparing different centrifugation protocols for PRF production, including L-PRF and H-PRF, reported relatively higher effects on the viability, proliferation, and adhesion of primary human dermal fibroblasts compared to osteoblasts. The very different methodologies employed impair the comparisons of these studies, but it is possible that the size of the effects of PRF on fibroblasts may be influenced by their tissue of origin, as fibroblasts from different anatomical sites may have distinct phenotypic and functional characteristics.

In the selected studies, different biological markers were observed that point to the molecular effects of PRF in cell differentiation and mineralization events (Table 2), depending on the cell type, including the expression and secretion of alkaline phosphatase, sialoproteins and sialophosphoproteins, type 1 collagen, osteocalcin, ostepontin, bone morphogenetic protein-2 (BMP-2), and the regulation of the transcription factors osterix and Runx2, which will be further discussed below.

Table 4 shows that a considerable proportion of the selected studies (n = 19, 25%) investigated the association of PRF with other compounds or materials in order to produce bioactive composites for different applications, which is a trend in the development of advanced, smart materials. These associations, which will be further discussed, include polymers (polycaprolactone meshes, chitosan/gelatin, polyvinyl alcohol/sodium alginate composites, PEG/PLGA copolymers), cements (MTA), calcium phosphates (nHA radiopacifiers, BCP, TCP), and allogenic or xenogeneic materials, including dentin chips and bovine bone substitutes. The myriad of methodologies and proposals impair the comparison of the relative performance of these materials between studies, and only a few of them included intra-study comparisons. Most of the studies indicated that the associations contribute to achieving the specific expected outcome, with increased cell response to PRF-containing composites, either by increased attachment, proliferation, or differentiation. As an exception, the study by Kyyak et al. [52] presented comparative evidence that the association of i-PRF with a xenogenic bone substitute material (XBSM) is less bioactive, promoting lower osteoblastic activity than allogenic bone substitute material (ABSM) associated with i-PRF. Nevertheless, further studies by Kyyak et al. [53] indicated that sintering XBSM samples under high temperatures could increase osteoblast viability and metabolic activity. Furthermore, Nguyen et al. [64] described that XBSM associated with other PRF types (A-PRF) enhanced human ligament stem cell proliferation and migration, even without sintering. 

## 4. Discussion

### 4.1. Protocols for PRF Production and Preservation

As indicated in Table 3, apart from the classical Choukrun’s L-PRF, other production protocols were also identified in the selected studies. These platelet concentrates differ not only in their preparation methods but also in mechanical properties, degradation rates, and growth factor release profiles, resulting in possible differences in the response of mineralizing cells after exposure. In this regard, a few studies compared such differences, with interesting findings that will be discussed below.

The study by Marchetti et al. [62] compared L-PRF with two other autologous biomaterials: concentrated growth factors (CGFs) and autologous platelet gel (APG). The authors reported stronger stimulation of the proliferation of human periodontal ligament fibroblasts (HPLFs) by CGFs compared to L-PRF and correlated these effects with the different secretion profiles of growth factors by these materials.

Verboket et al. [77] used the low-speed centrifugation concept, reducing the centrifugal force in the production of L-PRF to produce a membrane with a higher concentration of cells and growth factors. The study compared the protocols of PRF medium-RCF (1300 RPM for 8 min) and PRF low-RCF (700 RPM for 3 min). Although there was significant heterogeneity between the protocols to produce platelet aggregates and experimental design, the positive effects observed on proliferation, differentiation, and mineralization were rather similar between the protocols.

Fernandez-Medina et al. [34] compared the biological activity of A-PRF and i-PRF, along with two PRP protocols. After 21 days, i-PRF induced superior mineralization by human primary osteoblasts compared to the other materials, while A-PRF presented a negative impact at high concentrations, which was related to increased cytotoxicity. Despite its low content in growth factors, the author concluded that i-PRF was the best candidate for bone tissue engineering applications and was probably related to the prolonged release of BMP-2 by this material. Esmaeilnejad et al. [33] also investigated the effects of A-PRF, this time compared to classic L-PRF, on the cellular activity of MG-63 osteosarcoma-derived osteoblasts. Their findings indicate that L-PRF induced higher proliferation than A-PRF, while the latter was only capable of inducing in vitro mineralization at the employed experimental conditions, suggesting positive but very different effects of these materials. The study by Kosmidis et al. [50], on the other hand, investigated the effects of A-PRF, L-PRF, and i-PRF on the osteogenesis of the human osteoblast-like U2OS cell line. Similar to the findings by Esmaeilnejad et al. [33], A-PRF induced more mineralization and calcium production. i-PRF induced more ALP activity, suggesting it has the potential to enhance early cell differentiation.

Lourenço et al. [13] suggested using swing-out rotors instead of fixed-angle centrifugation to produce a product similar to L-PRF. According to some authors, the process known as H-PRF (platelet-rich fibrin produced through horizontal centrifugation) allows for a greater number of live cells and growth factors to be distributed more evenly in the final product. Al-Maawi et al. [18] compared the effects of PRF products produced by fixed-angle and horizontal centrifugation over osteoblast behavior, identifying very similar effects on proliferation and viability, with increased cell adhesion in the fixed-angle group. These results indicate that very similar materials may be produced with different centrifuge types, as long as the g-force is standardized.

The study by Li Q et al. [55] included physical modifications in the PRF protocol to increase shelf life, such as freeze-drying membranes. Their results indicated that the proliferation and migration of periodontal progenitors were increased with exposure to lyophilized PRF compared to fresh PRF, which was probably due to increased porosity and the release of growth factors from the processed membranes. Kardos et al. [47] also analyzed PRF membranes in different presentations (fresh, frozen, and freeze-dried) compared different centrifugation protocols (1700 RCF in 5 min and 8 min) and the use of modified tubes (single-syringe closed system—hypACT Inject Auto). Living cells were observed only in fresh PRF membranes, while freezing induced, as expected, the disruption of leukocytes embedded in the PRF membrane. However, MSCs were reported as proliferating even faster over freeze-thawed PRF than over fresh samples, suggesting the adequacy of such a procedure. 

Liu et al. [59] proposed the combination of fresh and lyophilized PRF at different ratios, tailored for different delivery rates of GFs in tissue healing. Their findings indicate a significant increase in proliferation and differentiation of BMSCs exposed to eluates of different combinations, with the best results achieved a fresh/lyophilized PRF ratio of 1:1. A subsequent study by the same group [60] proposed the use of a natural crosslinker agent, genipin, derived from gardenia flowers, to increase the stability and controlled release of growth factors by lyophilized PRF. Genipin-modified lyophilized PRF presented better biomechanical properties, slower biodegradation, and sustained release of growth factors, promoting the proliferation of pulp stem cells from human exfoliated deciduous teeth (SHEDs).

Isobe et al. [44] added the anticoagulant formulation acid citrate dextrose solution-A (ACD-A) in blood samples, with the objective of enabling its storage for later production of PRF. Its coagulation was obtained by the addition of CaCl_2_ (showing similarities with the protocol of PRP production), without a significant reduction in its bioactivity. This method tends to improve the flexibility of successful PRF preparations, and the quality of membranes prepared from whole blood samples were stored for up to 2 days.

### 4.2. Association of PRF and Other Materials/Compounds

Several of the selected studies investigated the association of PRF with other agents, including biomaterials, scaffolds, bioactive compounds, and ions, as described in Table 4. Irastorza et al. [43] investigated the use of L-PRF as a biomimetic coat of titanium implant surfaces, aiming for improved osteointegration. When combined with hDPSCs (human dental pulp stem cells), the material induced both proliferation and osteogenic differentiation of stem cells. The study by Steller et al. [73] also assessed the use of PRF coating over titanium implants, this time to reverse the negative effects of a bisphosphonate (zoledronic acid) over osteoblast attachment to the implant surfaces. Indeed, zoledronic acid led to a decrease in osteoblast adherence onto the implant surface, but it was reversed by a previous coating with L-PRF, suggesting that PRF may contribute to bone apposition in dental patients undergoing bisphosphonate treatment. Furthermore, L-PRF acted directly on the viability, migration, and proliferation of osteoblasts and fibroblasts treated with zoledronic acid [74].

Song et al. [72] explored the effectiveness of 3D-printed biphasic calcium phosphate/PVA scaffolds combined with PRF using a straightforward low-temperature method. These scaffolds demonstrated impressive biological activity and biocompatibility in vitro. When implanted into critical bone defects in a rabbit’s radius, the inclusion of PRF encouraged proper bone regeneration and repair by providing osteoconductive and osteoinductive stimuli [72]. Similarly, Sui et al. [75] proposed the production of 3D-printed L-PRF scaffolds composed of chitosan (CS)–hydroxyapatite (HAP) associated with lyophilized PRF. MC3T3-E1 murine preosteoblasts presented increased proliferation over the composite scaffold after association with PRF. The proliferation of preosteoblasts into the scaffolds increased with the release of GFs, indicating that L-PRF remains bioactive even after 3D printing. Zhang et al. [86] fabricated a complex, multifunctional triple-layered composite scaffold including polycaprolactone/gelatin (PG) nanofiber films made by electrospinning, chitosan/poly (γ-glutamic acid)/hydroxyapatite (CPH) hydrogels, and platelet-rich fibrin (PRF). The resulting scaffold presented induced the proliferation of both fibroblasts and bone mesenchymal stem cells (BMSCs) and also induced osteogenic differentiation in the latter. Other calcium phosphates, such as tricalcium phosphate (TCP), also presented interesting outcomes after association with PRF, as reported by Wong et al. [81], where the composite presented a controlled release of bioactive factors with the increase in osteoblast attachment, cell proliferation, migration, and ECM formation. 

Dentin, known for its bone-inducing properties, fuses and is gradually replaced by bone when grafted into it. This is likely due to its osteoinductive properties, biocompatibility, and BMP content [93]. In a study by Ji et al. [45], after seven days of coculture with PRF and a treated dentin matrix, BMSCs exhibited increased expression of BSP and OPN mRNA, while PDLSCs showed higher expressions of BSP, OPN, and OCN. Mahendran et al. (2019) [94] combined PRF with dentin chips and nanohydroxyapatite to enhance radiopacity, creating a biocompatible structure that promoted cell proliferation as a mitogen. Girija and Kavitha [39] compared the combination of PRF with 50 wt% of radiopacifier nanohydroxyapatite (nHA) or with 50 wt% dentin chips and their effects on odontoblastic differentiation. While both materials increased the expression of dentin sialophosphoprotein (DSP) and dentin matrix protein-1 (DMP-1), there were two important extracellular matrix proteins involved in the differentiation and mineralization of human dental pulp cells (HDPCs), and exposure to PRF + 50 wt% nHA induced more mineralization nodules in these cells. 

Zheng et al. [88] found that when developing a combination of PRF with a poly-polyethylene glycol (PEG)-PLGA copolymer, the hydrogel was evenly distributed on the inner surface of the PRF scaffolds. The hydrogel did not impact the inherently high porosity of the PRF scaffolds. A system containing nHA/PLGA/Gel/PRF allowed for a slow and sustained release of PRF-derived growth factors, leading to increased adhesion and the proliferation of MG63 human osteoblasts.

HDPCs treated with mineral trioxide aggregate (MTA) and PRF extracts exhibited a significantly increased expression of dentin sialoprotein and dentin matrix protein-1 along with enhanced ALP activity and mineralization compared to MTA or PRF treatment alone. The MTA and PRF extracts together activated bone morphogenic proteins (BMPs), while the BMP inhibitor LDN193189 diminished dentin sialophosphoprotein and dentin matrix protein-1 expression, ALP activity, and mineralization enhanced by MTA and PRF treatment [83].

In the study by Blatt et al. [22], four different bone substitute materials (allogeneic, alloplastic, and two of xenogeneic origin) were associated with a combination of A-PRF and i-PRF. The addition of PRF increased cell proliferation and migration for all bone substitutes, but only the allogeneic and alloplastic materials significantly increased Runx2 expression in human osteoblasts. On the other hand, bone morphogenic protein was expressed significantly higher when xenogeneic material was combined with PRF, suggesting that the biofunctionalization of bone substitutes with PRF might improve their performance, even for materials of different origins.

### 4.3. Cell-Type Related Effects of PRF

PRF possesses significant proliferative potential due to its fibrin structure, which contains live leukocytes and activated platelets. This promotes the continuous release of growth factors such as FGFs, PDGFs, TGF-beta1, and VEGFs. These factors act through specific signaling pathways, including MAPK and PI3K/Akt, which modulate gene expression and osteoblast proliferation and survival [94].

Gingival stromal progenitor cells (GSPCs) are a population of mesenchymal stem cells derived from the gingival connective tissue that have been shown to possess multipotent differentiation capacity, including osteogenic, adipogenic, and chondrogenic lineages. The study by Nugraha et al. [67] investigated the effects of PRF on the osteogenic differentiation of GSPCs, identifying a role of the Cbfa1/Sox9 expression ratio in this process, as it positively correlated with the osteogenic markers and the mineralization of GSPCs. Later, another study by this group [69] showed that PRF also increased the expression of aggrecan, a chondrogenic differentiation marker that has a significant role in the early stage of osteogenic differentiation of gingival stem cells, which may contribute to accelerating bone remodeling with an increased expression of alkaline phosphatase and osteocalcin [67,68]. 

Bone marrow stem cells (BMSCs) are multipotent progenitor cells with regenerative potential for various tissues, including bones [95]. In the selected studies, these cells exhibited increased proliferation when combined with PRF, irrespective of direct or indirect exposure [12,28,30,38,47,60]. These results not only provide evidence for the potential clinical success of PRF but also lay the foundation for its further use in bone tissue engineering. Although BMSCs are widely used in cell therapy, one of the main challenges is ensuring their efficient migration and homing to target tissues following systemic or local administration. The proliferative effects of PRF on BMSCs, combined with their other properties, such as biocompatibility, biodegradability, mechanical strength, porosity, and potential for vascularization [96], could help overcome the limitations of conventional injection-based delivery of BMSC single-cell suspensions and enhance their retention and engraftment in injured tissues. The results by Cheng et al. [25] regarding the exposure of BMSCs to L-PRF also provided some insights into its effects when combined with environmental factors, such as exposure to hydrostatic pressure, a mechanical stimulus that plays an important role in bone formation. In this sense, the co-culture of BMSCs with L-PRF reversed the activation of Wnt/Ca^2+^ signaling in BMSCs under hydrostatic pressure, with an increased expression of chondrogenic differentiation markers, suggesting a modulation of the differentiation pathways by the released growth factors from PRF.

Human adipose stem cells (hASCs) are a type of mesenchymal stem cell (MSC) that can be isolated from adipose tissue and have the potential to differentiate into various cell types, including osteoblasts. This complex process involves multiple factors such as growth factors, signaling pathways, transcription factors, and epigenetic modifications [97]. hASCs are attractive for regenerative medicine applications because they are autologous, abundant, and easily accessible. However, various factors like age, disease, and culture conditions can influence the proliferation and differentiation capacities of hASCs. Consequently, enhancing hASC proliferation is crucial for their clinical use. Human ASCs exhibited increased proliferation when exposed to different concentrations of PRF eluates [54]. Platelet-derived growth factor (PDGF-BB), one of the main growth factors secreted by PRF, has been shown to be a potent mitogen for hASCs [97]. It induces multiple signaling pathways such as ERK1/2, PI3K/Akt, and JNK, which regulate various cellular processes, including cell cycle progression through the expression of cyclins D1 and E. Previous studies have demonstrated that hASC proliferation may be blocked by inhibitors of the PDGF receptor tyrosine kinase, ERK1/2, and Akt, but not by a p38 inhibitor. These results suggest that the proliferation of hASCs through platelet concentrates, like PRF, may be mediated by the PDGF-BB-induced activation of ERK1/2, PI3K/Akt, and JNK signaling pathways.

Apical papilla stem cells (SCAPs) are mesenchymal stem cells found at the root tip of developing teeth. They play a crucial role in root formation and dentin–pulp complex regeneration. SCAPs secrete various bioactive factors that can modulate their microenvironment and influence tissue repair and regeneration [86], including some involved in forming bone-like tissue. The present search revealed that SCAPs exhibited increased proliferation when exposed to PRF eluates [23,37]. These findings are promising for developing stem cell-based bone therapies combining PRF and SCAPs, with the feasibility of transplantation in treating bone defects already demonstrated in animal models.

Another type of mesenchymal stem cell investigated in association with PRF is one that resides in the periodontal ligament, a connective tissue that anchors the tooth to the alveolar bone. Periodontal ligament stem cells (PDLSCs) have demonstrated osteogenic potential, meaning they can differentiate into osteoblasts and produce the bone matrix, playing a vital role in periodontal tissue regeneration and bone repair [98]. These cells exhibited proliferation when co-cultured with PRF [68,80] or after exposure to eluates [70,80]. These effects may contribute to the reported success in improving the regenerative potential and healing of PDL tissues presented by PRF in cases of late dental replantation or intraosseous defects.

Dental pulp stem cells (DPSCs) are also mesenchymal stem cells present in the dental pulp tissue of human teeth and have the potential to differentiate into osteoblasts, odontoblasts, adipocytes, and neural cells. DPSCs have been shown to promote wound healing, bone regeneration, nerve regeneration, and pulp regeneration in animal models and clinical trials. DPSCs offer a promising source of autologous stem cells that can be used for tissue engineering and the cell therapy of dental and oral diseases. In this sense, several selected studies assessed the exposure or association of DPSCs with PRF. Huang et al. [42] showed that L-PRF increases the proliferation and differentiation of human DPSCs [38]. The study by Bagio et al. [19] evidenced that A-PRF induces an increase in the expression of VEGF-A by these cells, which is an important factor in the angiogenesis process in dental pulp regeneration. More recently, Zhang et al. [87] have shown that i-PRF eluates induce a dose-dependent increase in the proliferation of hDPSCs and the expression of osteo-/odontoblastic differentiation markers and key proteins in the Notch signaling. This is a key pathway that regulates the fate and function of DPSCs, modulating the balance between self-renewal and differentiation, along with their angiogenic properties for tissue regeneration and repair. Lo Monaco et al. [61] exploited these effects by proposing the association of DPSCs with L-PRF for the treatment of osteoarthritis, as these cells can undergo chondrogenic differentiation and secrete growth factors associated with tissue repair. Indeed, the association with PRF promoted in vitro chondrogenesis and stimulated the survival of articular chondrocytes.

Osteoblasts are bone-forming cells that secrete extracellular matrix proteins supporting bone tissue formation and mineralization. These cells are derived from various progenitor populations, such as mesenchymal stromal/stem cells (MSCs), with their differentiation influenced by molecular mechanisms including signaling pathways, transcription factors, and epigenetic events [11]. Osteoblasts produce and respond to the main growth factors stimulating bone formation and are typically released by PRF and its derivatives. The present search found evidence that primary human osteoblasts and murine MC3T3-E1 preosteoblasts exhibit strong proliferative responses to PRF [12,22,33,35,43,45,48,49,76,80]. However, it is important to note that human osteosarcoma cell lines SaOS, MG63, and U2OS also showed consistent proliferative behavior after exposure to PRF [17,23,27,29,55,60,61,72,77,83]. This finding warrants further assessment and should be considered by clinicians for its potential risk of stimulating cancer cell growth and dissemination by creating a favorable niche for tumor development.

Due to the good cell response to PRF, some authors proposed the use of this material as a scaffold for bone cell therapy. It includes the proposal by Gassling et al. [35,38] for the use of PRF for periosteal tissue engineering, who reported an increased proliferation of periosteal cells [35] and human osteoblasts [38] over PRF compared to collagen scaffolds (Bio-Gides). As expected, PRF also promoted increased expression of osteogenic markers in osteoblasts compared to commercial collagen scaffolds.

In summary, the in vitro evidence from the selected studies highlights a robust effect on the proliferation of different cell types as an essential aspect of the clinical potential of PRF in treating bone and other mineralized tissues.

### 4.4. The Molecular Effects of PRF on Differentiation and Mineralization

**Figure 3:**
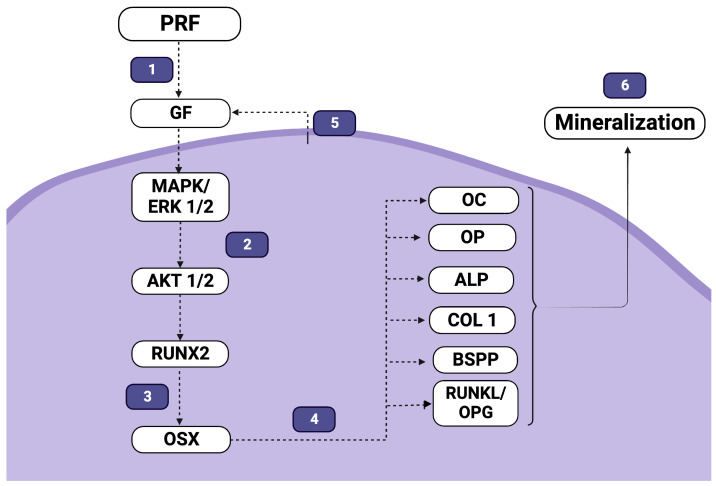
Main biological events involved in the response of mineralizing cells to PRF, according to the literature evidence. Several growth factors and important mediators are released in the medium by PRF (1), many of which are able to activate mitogen-induced signaling pathways (2), which are known to modulate the expression of transcription factors, such as Runx2 and osterix, directly involved on bone cell differentiation (3). These transcription factors are responsible for the altered expression and secretion of different proteins responsible for the formation of the mineralized matrix and the control of osteoclastogenesis (4). Furthermore, exposure to PRF may induce increased secretion of growth factors, such as BMP-2, VEGF, or IGF (5), indicating that exposed cells may also be activated by paracrine regulation in response to PRF. All of these processes result in increased nodule formation and, therefore, biological mineralization (6). In the figure, an arrow indicates activation/stimulation, while a perpendicular bar indicates inhibition/suppression.

Bone regeneration is a complex process involving the interaction of cells, growth factors, and the extracellular matrix. Clinical studies have described PRF as effective in oral and maxillofacial bone regeneration, increasing the amount of bone formation following procedures such as sinus floor elevation [99]. The molecular effects of PRF on the differentiation and mineralization of bone cells may involve various intracellular signaling pathways, as evidenced by the selected studies in this review and the summary in Figure 3.

The mitogen-activated protein kinase (MAPK) pathway is a cell signaling route that regulates numerous biological processes, including cell differentiation. Among the MAPKs, ERK1/2 plays a crucial role in osteoblast differentiation by regulating the expression of osteogenesis-related genes [90]. The phosphorylation of ERK was increased by exposure to PRF in the osteosarcoma-derived osteoblast cell line U2OS [23,100]. Consequently, the expression of osteoprotegerin (OPG) was upregulated by PRF, while the expression of the receptor activator of NFkB ligand (RANKL) was not significantly altered. These results suggest that PRF could inhibit osteolytic activity by modulating osteoclastogenesis through the control of the OPG/RANKL ratio in osteoblasts [23,100].

The Runt-related transcription factor 2 (Runx2) is a key transcription factor that regulates the expression of genes involved in osteoblast differentiation and function. Research has demonstrated that various members of the MAPK family, including ERK1/2 and p38, can phosphorylate Runx2 at specific serine residues, enhancing its transcriptional activity and promoting osteogenic gene expression [101]. As a result, ERK1/2 and Runx2 are connected by a positive feedback loop that stimulates osteoblast differentiation and bone formation. In selected studies, a significant increase in the expression and/or phosphorylation of Runx2 was detected after exposure to platelet-rich fibrin (PRF) in different cell types [30,51,52,53,66,74,86]. The studies by Wang et al. [80,81] confirmed, with both human bone marrow and rabbit mesenchymal stem cells, that exposure to PRF preparations increases proliferation, the expression of osteogenic markers, and in vitro mineralization concomitant to the upregulation of the ERK 1/2 signaling pathway.

Additional evidence of these regulatory pathways comes from investigations of osterix (Osx), a zinc-finger transcription factor essential for osteoblast differentiation and bone formation in bone homeostasis [102]. Osx is a downstream target of Runx2 and regulates several target genes involved in osteoblast differentiation, such as Col5a1, Col5a3, and connexin43 (Cx43). Osx is controlled by multiple signaling pathways, including BMP, Wnt, FGF, and ERK1/2, which modulate its transcriptional activity and stability [103]. Data from a study by Li et al. [56] suggest that osterix levels significantly increase in periodontal ligament stem cells treated with PRF and IGF-1 at 14 days compared to the control group (*p* < 0.01). Furthermore, by the third day of exposure, the expression of genes controlled by Osx was upregulated in the PRF-exposed group. These findings imply that these transcription factors contribute to the stimulation of osteoblast differentiation by PRF, potentially through the activation of MAPKs in response to growth factors released by this autologous biomaterial.

The overexpression of Osx and Runx2 after exposure to PRF could potentially impact several essential proteins for osteoblast maturation and mineralization, whose expression is regulated by these factors, including collagen type-I a1 (Col1a1), osteonectin, osteopontin, bone sialoprotein, and osteocalcin. Osteocalcin is responsible for fixing calcium and hydroxyapatite in the extracellular matrix, contributing to the effective mineralization that occurs in bone tissue [104]. Molecular analyses through real-time PCR have shown a significant increase in RNA expression for osteocalcin in various mineralizing cells exposed to PRF [26,43,63,68,73,79]. These findings were reinforced by a functional assessment by western blotting, indicating increased levels of this protein in periodontal ligament stem cells, which is associated with increased bone formation in a rat in vivo model [30].

Different studies have reported that PRF also induces increased expression of dentin sialoprotein (DSP) and dentin sialophosphoprotein (DSPP) [24,77], which are enhanced in the presence of lipopolysaccharide [47]. The results by Hong et al. [41] suggest that this effect is dependent on the duration of exposure, as the expression level of DSPP was downregulated after incubation in PRF for 7 days and then significantly increased after 14 days of incubation. Bone sialoprotein (BSP) is also reported to increase when cells are exposed to PRF, primarily around 14 to 21 days [24,30,39,43,63]. Only human periodontal ligament stem cells had DSPP downregulated when co-cultured with PRF over extended experimental periods [81]. These results are important since this non-collagenous protein plays a crucial role in the biomineralization of hard tissues, such as bones and teeth, inducing the formation and growth of hydroxyapatite crystals in the extracellular matrix [104].

Osteopontin is another phosphorylated glycoprotein secreted into the mineralizing extracellular matrix by osteoblasts during bone development. Although osteopontin is an osteogenic marker that does not affect the cellular development of osteoblasts in vitro, it impacts the mineralization of bone tissue [105]. Various studies have shown that this osteogenic marker is present and highly expressed after exposure to PRF in osteoblasts [20], dental pulp cells [79], and mesenchymal stem cells (MSCs) [73]. Real-time PCR revealed that bone marrow-derived mesenchymal stem cells (BMSCs) cultured on printed BCP/PVA/PRF scaffolds expressed significantly higher levels of osteopontin on days 7 and 14 compared to those cultured on other scaffolds (*p* < 0.05) [66]. Verboket et al. [66] observed a higher expression in bone marrow cells exposed to a medium and low RCF PRF, indicating that these protocols did not affect the expected effects of the material on the expression of matrix proteins.

The increase in collagen gene expression induced by PRF was reported in several studies [27,69,75,78,79], typically around the 14th to the 21st day of exposure. Zhao et al. [88] observed slight upregulation by PRF in a dose-dependent manner after 14 or 21 days of culture (*p* < 0.01). In contrast, Ji B et al. [45] and Verboket et al. [77] did not observe a significant difference in the expression of Col-III, the primary type of collagen in bones, between the control group and the test group.

ICAM-1 (intercellular adhesion molecule 1) is a protein that mediates cell–cell interactions and plays a role in inflammation and immune responses. ICAM-1 is expressed by various cell types, including osteoblasts, and it may have different effects depending on the context [98]. In healthy individuals, ICAM-1 may facilitate osteoblast contact with lymphocytes, enhancing mineralization and bone formation through the downregulation of TGF-β1 (transforming growth factor beta 1), a negative regulator of osteogenesis. In pathological conditions, such as osteoarthritis and osteoporosis, ICAM-1 expression by osteoblasts may be increased by proinflammatory cytokines and contribute to bone resorption and loss of bone mass. ICAM-1-positive osteoblasts can adhere to osteoclast precursors and stimulate their differentiation and activation through the osteoclastogenic factor RANKL [106]. Moreover, ICAM-1-positive osteoblasts may have impaired proliferation and differentiation potential due to cell cycle arrest [99]. When exposed to both regular and low-speed PRF, Kang et al. [46] and Verboket et al. [77], respectively, observed a high expression of ICAM-1 in mesenchymal stem cells, including in the presence of lipopolysaccharide to stimulate inflammation. On the other hand, Dohle et al. [29] reported that PRF appears to have no effect on the expression of the ICAM-1 protein by exposed primary human osteoblasts. This is an important finding since PRF is described as releasing considerable amounts of proinflammatory cytokines [13,107].

Alkaline phosphatase (ALP) is located on the outer surface of the cell membrane of osteoblasts. ALP is an early marker of osteoblast differentiation that indicates the transition of osteoprogenitor stem cells to immature preosteoblasts [102]. It plays an essential role in osteoid formation and mineralization and is recognized as a nonspecific marker of bone formation and osteoblast activity. ALP also regulates RUNX2, a master transcription factor in osteoblasts, through a positive feedback loop that modulates osteoblast differentiation. The present review identified several different studies assessing the effects of exposure to PRF on the expression of ALP, with increased levels evidenced from 14 to 21 days of culture [25,26,29,39,52,56,64,83,85]. The study by Woo et al. [83] observed that the effects of PRF associated with MTA on ALP and the in vitro mineralization of human dental pulp cells were impaired by pretreatment with a BMP inhibitor (LDN193189), indicating the participation of specific growth factors in the effects of PRF on ALP.

Osteoblast and bone cell activity are regulated by various growth factors, many of which are released by platelet-rich fibrin (PRF) membranes. Examples of these growth factors include transforming growth factor-beta (TGF-beta) and fibroblast growth factors (FGFs). Osteoblasts themselves can produce and release growth factors that regulate the differentiation and activity of other bone cells, such as osteoclasts and osteocytes. These factors include RANKL/OPG, M-CSF, VEGFs, PDGFs, and BMPs [12]. These growth factors can act in paracrine or endocrine manners, influencing bone and systemic metabolism by promoting or inhibiting the differentiation and function of various bone cells. When exposed to PRF, osteoblasts engage in a complex cellular communication system involving both the exposure and secretion of growth factors that impact bone tissue, as evidenced by selected studies.

TGF-beta, a member of the TGF-beta superfamily of cytokines, regulates numerous cellular processes such as proliferation, differentiation, migration, and apoptosis. TGF-beta plays a significant role in bone formation and remodeling by influencing the balance between bone-forming cells (osteoblasts) and bone-resorbing cells (osteoclasts) [108]. TGF-beta stimulates osteoblastic differentiation and activity by activating canonical and non-canonical signaling pathways and increasing the expression of osteogenic genes like Runx2, osterix, and collagen I in osteoblasts. Conversely, TGF-beta inhibits osteoclastogenesis and osteoclastic activity by modulating the interaction between osteoblasts and osteoclasts through OPG/RANKL, BMPs, and Wnt proteins, thereby affecting both aspects of the bone remodeling cycle.

Studies have shown that osteoblasts exposed to PRF demonstrate increased production of TGF-beta1. For instance, Saos-2 osteosarcoma-derived osteoblasts exposed to PRF exhibited significantly higher TGF-beta1 production [105]. Rat calvaria osteoblasts exposed to PRF showed a time-dependent increase in TGF-beta1 production [48]. Human alveolar bone marrow stem cells treated with PRF extracts exhibited increased TGF-beta1 levels at 24 h [46]. A study by Kim et al. [49] compared the release of TGF-beta1 associated with PRF in osteoblast cultures exposed to PRF membranes produced by different protocols. The results indicated that the secreted levels of the growth factor were significantly higher in low-RCF PRF than in medium-RCF PRF (*p* < 0.05) [76]. However, lyophilized PRF samples only slightly increased the release rate of TGF-beta1 in another osteosarcoma cell line, MG63 [85].

Bone morphogenetic protein 2 (BMP-2) is a multifunctional protein belonging to the TGF-beta superfamily. It plays a crucial role in bone formation by stimulating the differentiation of mesenchymal stem cells (MSCs) into osteoblasts. Osteoblasts and osteocytes constitutively secrete BMP-2, which has been used as a therapy for bone fractures and diseases, such as osteoporosis, due to its osteogenic potential [14]. Rabbit mesenchymal stem cells exposed to PRF demonstrated a significant upregulation of BMP-2 mRNA expression, reaching levels that stimulate in vitro osteogenic differentiation [96]. Combining 1 mg/mL MTA with 1.25% PRF extracts increased BMP-2 expression in human dental pulp cells compared to PRF extracts alone, while MTA treatment alone showed no release [83]. BMP-2 expression was also higher in the co-cultures of i-PRF and primary osteoblasts [29].

Saos-2 cells exposed to PRF exhibited noticeably higher peaks of insulin-like growth factor-1 (IGF-1), especially compared to fibroblast cultures [105]. Human alveolar bone marrow stem cells exposed to PRF extracts showed a high expression of IGF-1 [107], similar to MG63 osteosarcoma cells and primary osteoblasts [66,76]. These findings are significant since IGF-1 plays a crucial role in regulating osteoblast function and development [108]. IGF-1 initiates a complex signaling pathway involving the PI3-K/PDK-1/Akt and Ras/Raf/MAPK pathways, which stimulate cell function and/or survival. IGF-1 also influences osteoclastogenesis by regulating RANKL and RANK expression.

The available data suggest that most of PRF’s effects on bone and mineralizing cells may be attributed to the release of specific biological mediators that activate signaling pathways related to cell proliferation, survival, and differentiation. Investigating the release of growth factors, cytokines, and chemokines by PRF membranes could help understand these effects. However, only a few selected studies have detected such release in their in vitro assessments. Zhao Y-H et al. [88] observed a time-dependent decrease in VEGFs, IGF-1, and EGF release from PRF membranes within five days. In contrast, Dohle et al. [29] found the highest concentration of VEGFs in osteoblast supernatants mixed with PRF cultured for seven days, confirmed by relative gene expression of VEGFs after 24 h of osteoblast monocultures. Isobe et al. [44] observed that PDGF-BB concentration was significantly reduced in extracts from PRF membranes made from stored blood compared to fresh samples, indicating a potential drawback of long storage. Zheng et al. [89] observed a sustained release of PDGF, IGF-I, and TGF-B1 for up to four weeks when combining nHA/PLGA/gel with lyophilized PRF, which positively affected MG63 cell mineralization. Studies evaluating platelet-derived growth factor (PDGF AA, AB, or BB) reported variations in the day of the highest in vitro release [27,38,43], ranging from the 1st to the 14th days of exposure. This suggests that the choice of extraction time may impact the observed effects of PRF treatment.

## 5. Final Considerations

Due to the considerable relevance of the theme for regenerative medicine and dentistry, several narrative and systematic reviews have been published issuing the clinical and biological effects of PRF. Many of these reviews are focused on the level of evidence supporting the use of PRF in regenerative dentistry and oral and maxillofacial surgery by assessing randomized clinical trials [2], including data indicating that PRF significantly improves bone tissue regeneration [109,110], regardless of recent reviews on preclinical studies that failed to identify such effects on animal models [111]. Despite any clinical controversies, only a few literature reviews visited the molecular and cellular evidence of PRF effects and are usually restricted to smaller groups of in vitro studies focusing on specific protocols, such as i-PRF [112]. An exception is the interesting review by Strauss et al. [113], who assessed in vitro evidence of the biological effects of PRF in cells from different tissues published up to 2018. However, unlike the present review, that study was not focused on bone and mineralized tissues and had a limited reach, as it was based on data retrieved from a single database (Medline). Therefore, more than an update on the literature, the present scoping review represents the most comprehensive mapping of the available evidence supporting the molecular mechanisms that set the basis of the PRF effects in the behavior of the cells of bones and mineralized tissues.

While this approach provided valuable insights, it also introduced some limitations. By focusing exclusively on PRF, we ensured the feasibility and comparability of this review but may have overlooked data on other similar platelet aggregates or protocols, such as Concentrated Growth Factors (CGFs). Additionally, the scope of this review may be limited by the interpretation or reporting of the data, as some studies may have omitted important information necessary for a comprehensive understanding of the results. Nevertheless, it is possible to state, through the raised data, that the literature consistently provides in vitro evidence that PRF, produced by different protocols or in combination with other biomaterials, influences the proliferation, differentiation, and mineralization of cells from bone and mineralized tissue. These effects involve well-known pathways of cell survival, the activation of transcription factors (Runx2, OSX), and the increased expression of various proteins, including osteopontin, osteocalcin, collagen, ALP, BSPs, RANKL/OPG, and growth factors like TGF-beta, PDGF, and BMP2. Different studies have associated these effects with specific growth factors released by PRF, suggesting that this is an important factor to consider in the development and improvement of these autologous biomaterials for the treatment of mineralized tissues, such as bones.

PRF is widely used in regenerative medicine and dentistry, and in vitro models represent only a small aspect of wound healing and bone regeneration. As such, it is not possible to guarantee that the reported results can be directly extrapolated to clinical settings. On the other hand, improved in vitro proliferation, differentiation, and mineralization of osteoblasts may represent interesting evidence in support of enhanced regenerative activity, osteoinductive properties, or enhanced osseointegration. Therefore, by gathering and analyzing information related to PRF and mineralizing cells, we can gain a better understanding of the mechanisms behind PRF’s impact on bone regeneration and optimize its properties and applications. This includes determining optimal concentrations, exploring associations with other bone graft materials or drugs, and developing new production protocols to improve clinical outcomes of this promising autologous material.

## Figures and Tables

**Table 1:** The search key employed in the three consulted databases.

DATABASE	Search Key
PubMed (https://pubmed.ncbi.nlm.nih.gov/, accessed on 14 August 2023)	(PRF OR “platelet rich fibrin” OR L-PRF OR i-PRF OR “Sticky bone” OR “concentrated growth factors” OR CGF) AND (Bone OR osteoblast* OR MSC OR “mesenchymal stem cell” OR “bone marrow” OR “Bone and bones” [mh] OR “bone cell” OR preosteoblast* OR Skeleton) AND (“in vitro” OR In Vitro Techniques” [mh] OR “Cell Lineage” [mh] OR “Cells, Cultured” [mh]).
Web of Science (https://www.webofscience.com/, accessed on 14 August 2023)	((PRF OR “platelet rich fibrin” OR L-PRF OR i-PRF OR “Sticky bone” OR “concentrated growth factors” OR CGF) AND (Bone OR osteoblast OR MSC OR “mesenchymal stem cell” OR “bone marrow” OR “Bone and bones” OR “bone cell” OR preosteoblast OR PDL OR “periodontal ligament OR mineralization) AND (“in vitro” OR “In Vitro Techniques” OR “Cell Lineage” OR “Cells, Cultured”)) Refined by DOCTYPE: (ARTICLE).
Scopus (https://www.scopus.com/search/form.uri?display=basic, accessed on 14 August 2023)	TITLE-ABS-KEY (prf OR “platelet rich fibrin” OR l-prf OR i-prf OR “Sticky bone” OR “concentrated growth factors” OR cgf) AND TITLE-ABS-KEY (bone OR osteoblast OR MSC OR “mesenchymal stem cell” OR “bone marrow” OR “Bone and bones” OR “bone cell” OR preosteoblast OR pdl OR “periodontal ligament OR mineralization) AND TITLE-ABS-KEY ((“in vitro” OR “In Vitro Techniques” OR “Cell Lineage” OR “Cell Culture”) AND (LIMIT TO (DOCTYPE, “ar”))).

**Table 2:** Critical appraisal of the selected studies, performed according to the ToxRTool [16].

Publication	Group I: Test Substance Identification (4)	Group II: Test System Characterization (3)	Group III: Study Design Description (6)	Group IV: Study Results Documentation (3)	Group V: Plausibility of Study Design and Data (2)	Total (18)
Al-Maawi et al., 2021 [17]	4	3	6	3	2	18
Al-Maawi et al., 2022 [18]	4	3	6	3	2	18
Bagio et al., 2021 [19]	3	3	6	3	2	17
Banyatworakul et al., 2021 [20]	4	3	6	3	2	18
Bi et al., 2020 [21]	4	3	6	3	2	18
Blatt et al., 2021 [22]	4	3	5	3	2	17
Chang, Tsai, and Chang, 2010 [23]	4	2	5	3	2	16
Chen et al., 2015 [24]	4	3	6	3	2	18
Cheng et al., 2022 [25]	4	3	5	3	2	17
Chi et al., 2019 [26]	4	3	5	3	2	17
Clipet et al., 2012 [27]	4	3	6	3	2	18
Dohan Ehrenfest et al., 2010 [28]	4	1	5	3	2	15
Dohle et al., 2018 [29]	4	3	6	3	2	18
Douglas et al., 2012 [30]	4	1	5	3	2	15
Duan et al., 2018 [31]	4	1	5	3	2	15
Ehrenfest et al., 2009 [32]	4	1	5	3	2	15
Esmaeilnejad et al., 2022 [33]	4	3	6	3	2	18
Fernandez-Medina et al., 2019 [34]	4	3	6	3	2	18
Gassling et al., 2009 [35]	4	1	5	3	2	15
Gassling et al., 2010 [36]	4	3	5	3	2	17
Gassling et al., 2013 [37]	4	3	6	3	2	18
Gassling et al., 2013 [38]	4	3	5	3	2	17
Girija and Kavitha, 2020 [39]	2	3	5	2	1	13
He et al., 2009 [40]	4	3	5	3	2	17
Hong, Chen, and Jiang, 2018 [41]	4	3	6	3	2	18
Huang et al., 2010 [42]	4	3	6	3	2	18
Irastorza et al., 2019 [43]	4	3	6	3	2	18
Isobe et al., 2017 [44]	4	1	6	3	2	16
Ji et al., 2015 [45]	4	3	6	3	2	18
Kang et al., 2011 [46]	4	3	6	3	2	18
Kardos et al., 2018 [47]	4	2	5	3	2	16
Kim et al., 2017 [48]	4	2	5	3	2	16
Kim et al., 2017 [49]	4	3	5	3	2	17
Kosmidis et al., 2023 [50]	4	3	6	3	2	18
Koyanagi et al., 2022 [51]	4	3	5	3	2	17
Kyyak et al., 2020 [52]	4	3	6	3	2	18
Kyyak et al., 2021 [53]	4	3	6	3	2	18
Li et al., 2013 [54]	4	2	6	3	2	17
Li et al., 2014 [55]	4	3	6	3	2	18
Li et al., 2018 [56]	4	3	6	3	2	18
Li et al., 2018 [57]	4	3	6	3	2	18
Liang et al., 2021 [58]	3	3	6	3	2	17
Liu et al., 2019 [59]	4	3	6	3	2	18
Liu et al., 2022 [60]	4	3	6	3	2	18
Lo Monaco et al. 2020 [61]	4	3	6	3	2	18
Marchetti, 2020 [62]	4	3	6	3	2	18
Moradian et al., 2017 [63]	4	2	5	3	2	16
Nguyen et al., 2022 [64]	4	3	6	3	2	18
Nie et al., 2020 [65]	4	3	6	3	2	18
Nugraha et al., 2018 [66]	4	2	5	3	2	16
Nugraha et al., 2018 [67]	4	2	5	3	2	16
Nugraha et al., 2018 [68]	4	2	5	3	2	16
Nugraha et al., 2019 [69]	4	2	5	3	2	16
Rastegar et al., 2021 [70]	4	3	6	3	2	18
Shah et al., 2021 [71]	4	2	4	3	2	15
Song et al., 2018 [72]	4	3	6	3	2	18
Steller et al., 2019 [73]	4	3	6	3	2	18
Steller et al., 2019 [74]	4	3	6	3	2	18
Sui et al., 2023 [75]	4	3	6	3	2	18
Thanasrisuebwong et al., 2020 [76]	4	2	6	3	2	16
Verboket et al., 2019 [77]	4	2	6	3	2	17
Wang et al., 2015 [78]	4	3	6	3	2	18
Wang et al., 2018 [79]	4	3	6	3	2	18
Wang et al., 2022 [80]	4	3	5	3	2	17
Wang et al., 2023 [81]	4	3	5	3	2	17
Wong et al., 2021 [7]	4	3	6	3	2	18
Wong et al., 2021 [82]	4	3	6	3	2	18
Woo et al., 2016 [83]	4	1	5	3	2	15
Wu et al., 2012 [84]	4	3	5	3	2	16
Yu et al., 2016 [85] Yu et al., 2023 [86]	4	3	6	3	2	18
Zhang et al., 2019 [87]	4	3	6	3	2	18
Zhang et al., 2023 [88]	4	3	5	3	2	17
Zhao et al., 2013 [89]	4	2	5	3	2	16
Zheng et al., 2015 [90]	4	1	5	3	2	15
Zheng et al., 2020 [91]	4	3	6	3	2	18

**Table 3:** The main biological information of the selected studies.

Publication	PRF Protocol	Mineralizing Cell Type	Exposure Time	Exposure Method	Biological Parameters	Results
Al-Maawi et al., 2021 [17]	A-PRF	Primary human osteoblasts	After 3 and 7 days	Eluate	VEGF; TGF-β1; PDGF, OPG, IL-8; OPN; ALP activity.	PRF produced according to the low-speed centrifugation concept, associated with a polymeric scaffold, had a significant effect on osteogenic markers of osteoblasts.
Al-Maawi et al., 2022 [18]	L-PRF and H-PRF	Primary human osteoblasts (pOBs)	24 h	Eluate	Cell adhesion.	Osteoblasts exposed to PRF produced with fixed-angle rotors presented higher adhesion than those exposed to PRF produced with variable angles.
Bagio et al., 2021 [19]	A-PRF	Human dental pulp stem cells	5, 12 and 24 h	Eluate	VEGF-A.	5% A-PRF extracts increased VEGF-A expression by hDPSCs.
Banyatworakul et al., 2021 [20]	L-PRF	Canine periodontal ligament cells	1, 3 and 7 days	Eluate	Proliferation; migration; in vitro mineralization.	PRF derived from Thai buffalo blood promoted the proliferation, migration, and increased mineral deposition in vitro of canine periodontal ligament cells.
Bi et al., 2020 [21]	L-PRF	Stem cells from the apical papilla (SCAP)	1, 3 and 5 days	Eluate	Proliferation; migration; in vitro mineralization; ERK; pERK; ALP; DMP-1.	PRF improved the proliferation, migration, and the osteo-/odontogenic differentiation of SCAPs by activating the ERK pathway.
Blatt et al., 2021 [22]	A-PRF and iPRF	Human osteoblasts (HOBs)	24 h	Co-culture	Cell viability; proliferation; migration; ALP; Col-I; BMP2; Runx2.	The combination of PRF with bone substitute materials increased the viability, early proliferation, and migration potential of human osteoblasts via Runx2 alkaline phosphatase, collagen, and BMP2.
Chang, Tsai, and Chang, 2010 [23]	L-PRF	Human osteosarcoma osteoblast-like cells (U2OS)	1, 3 and 5 days	Co-culture	Proliferation; p-ERK, RANKL; OPG.	PRF stimulated osteoblast proliferation with positive regulation of the expression of p-ERK and increased the secretion of OPG.
Chen et al., 2015 [24]	L-PRF	Canine dental pulp stem cells (DPSCs)	7, 14 and 21 days	Co-culture	Proliferation; ALP activity; ALP; DSPP; DMP 1; BSP.	PRF not only provides a well-organized scaffold for cell adhesion and migration but also induces DPSC proliferation and differentiation markers.
Cheng et al., 2022 [25]	L-PRF	Rabbit bone marrow mesenchymal stem cells (BMSCs)	Up to 28 days	Co-culture	Mineralization; adipogenic differentiation; aggrecan, Col-II; Sox9; b-catenin; P-GSK3b; CaMKII; PKC.	Co-culture with PRF reversed the activation of Wnt/Ca^2+^ signaling in BMSCs under hydrostatic pressure, with increased expression of chondrogenic differentiation markers.
Chi et al., 2019 [26]	Decellularized PRF	Bone marrow stem cells	every 24 h for 9 days	Cultured on PRF	Adhesion; proliferation; Col I; ALP; OPN; OCN; Runx-2.	Decellularized PRF combined with chitosan/gelatin scaffolds accelerate the attachment, proliferation, and osteogenesis-related marker expression of bone marrow stem cells.
Clipet et al., 2012 [27]	L-PRF	Human osteosarcoma osteoblast-like cells (Saos-2)	1 and 2 days	Eluate	Cytotoxicity; proliferation; Cell Cycle; cbfa1, Col1, OCN; OPN	Exposure to a PRF-conditioned medium increased the cell viability, proliferation, and expression of the late and early markers of osteogenesis.
Dohan Ehrenfest et al., 2010 [28]	L-PRF	Human bone mesenchymal stem cells (BMSCs)	7, 14, 21 and 28 days	Co-culture	Cytotoxicity; proliferation; ALP activity; mineralization; morphology (SEM).	Increased proliferation and differentiation of BMSC when exposed to Choukroun’s PRF.
Dohle et al., 2018 [29]	iPRF	Primary osteoblasts (pOBs)	1 and 7 days	Cultured on PRF	VEGF; ICAM-1; ALP.	The expression of E-selectin, ICAM-1, VEGF, and ALP was significantly higher in the co-culture of primary osteoblasts and outgrowth endothelial cells cultured in PRF in vitro, in addition to improving the angiogenesis process.
Douglas et al., 2012 [30]	L-PRF	Human osteosarcoma osteoblast-like cells (Saos-2)	3, 5 and 7 days	Co-culture	Cytocompatibility; migration.	PRF functionalized with ALP and induced to mineralization was not cytotoxic and promoted colonization by human osteoblasts.
Duan et al., 2018 [31]	L-PRF	Rat periodontal ligament stem cells (PDLSCs)	1, 2, 3, 4, 7 and 14 days	Cultured on PRF	Proliferation; BSP; OCN; Runx2; ALP activity.	PRF enhanced cell proliferation and the expression of osteogenic markers in rat PDLSCs.
Ehrenfest et al., 2009 [32]	L-PRF	Human maxillofacial osteoblasts	7, 14, 21 and 28 days	Co-culture	Proliferation; mineralization; ALP activity.	PRF stimulates the proliferation of several very different cell types, and the effects on osteoblastic differentiation are highly significant.
Esmaeilnejad et al., 2023 [33]	A-PRF and L-PRF	Human osteosarcoma osteoblast-like cells (MG-63)	24 and 72 h	Eluate	Proliferation; mineralization.	L-PRF increased proliferation, while A-PRF increased the in vitro mineralization of MG-63 cells.
Fernandez-Medina et al., 2019 [34]	A-PRF and I-PRF	Primary human osteoblasts	24 and 72 h	Eluate	Proliferation; migration; mineralization; cytokine release.	Cell viability and migration assays have demonstrated a detrimental effect when the concentration was ≥60. i-PRF demonstrated superior induction of mineralization. A negative impact of A-PRF was demonstrated at high concentrations.
Gassling et al., 2009 [35]	L-PRF	Human osteoblasts (HOBs) and osteosarcoma (Saos-2)	10 days	Co-culture	PDGF; IGF; TGF-Beta.	PRF exposure led to an increased secretion of growth factors by osteoblasts.
Gassling et al., 2010 [36]	L-PRF	Human periosteal cells	10 min, 1 h and 1 day.	Eluate	Cell viability; proliferation.	PRF appears to be superior to collagen (Bio-Gide) as a scaffold for human periosteal cell proliferation.
Gassling et al., 2013 [37]	Mg-enhanced and enzymatically mineralized PRF	Human osteosarcoma osteoblast-like cells (Saos-2)	1, 3 and 7 days	Co-culture	Cell viability; proliferation; morphology.	The enzymatic mineralization of PRF did not affect osteoblast viability and the proliferation on the membrane.
Gassling et al., 2013 [38]	L-PRF	Human osteoblasts	1, 5, 7 and 36 days	Eluate	Cell viability; proliferation, ALP activity.	The PRF membrane supports the proliferation of human osteoblast cells, in addition to being an adequate support for the cultivation of human osteoblasts in vitro.
Girija and Kavitha, 2020 [39]	PRF (undefined protocol)	Dental pulp cells	2 h, overnight	Eluate	IL-6; IL-8; DMP-1, DSPP, STRO-1; mineralization.	The addition of bioactive radiopacifiers into PRF has a synergistic effect on the stimulation of odontoblastic differentiation of HDPCs, inducing mineralization.
He et al., 2009 [40]	L-PRF	Rat calvaria osteoblasts	1, 7, 14, 21 and 28 days	Exudates	Proliferation; mineralization; ALP activity; cytokine release.	PRF released autologous growth factors gradually and expressed stronger and more durable effects on the proliferation and differentiation of rat osteoblasts than PRP in vitro.
Hong, Chen, and Jiang, 2018 [41]	Freeze-dried L-PRF	Apical papilla (SCAPs)	7 and 14 days	Membrane dissolved in 10 mL of DMEM	Proliferation; migration; morphology; differentiation (CD45, CD90, and CD146), mineralization; ALP; BSP; DMP-1; DSPP.	Freeze-dried PRF promotes the proliferation, migration, and differentiation of SCAPs
Huang et al., 2010 [42]	L-PRF	Dental pulp cells (DPCs)	0, 1, 3 and 5 days	Co-culture	Viability; proliferation; OPG; ALP activity.	PRF stimulates cell proliferation and the differentiation of DPCs by upregulating OPG and ALP expression.
Irastorza et al., 2019 [43]	L-PRF	Pulp stem cells	4 days	Co-culture	Mineralization; ALP activity; ALP; Col-I; Osteonectin; Runx2, OSX.	Osteoblastic differentiation from human pulp stem cells was achieved with a combination of biomimetic rough titanium surfaces (BASTMs) with autologous plasma-derived fibrin-clot membranes.
Isobe et al., 2017 [44]	L-PRF from stored (frozen) blood	Human periosteal cells	3 days	Eluate	Proliferation.	The quality of PRF clots prepared from stored whole blood samples is not significantly reduced and induced similar proliferation of periosteal cells as fresh PRF.
Ji et al., 2015 [45]	L-PRF	Periodontal ligament stem cells and bone marrow mesenchymal stem cells	1, 2, 3, 4, 5, 6 and 7 days	Transwell inserts	Migration; proliferation; BSP; OCN; OPN; Col-III.	The association of PRF and TDM (treated dentin matrix) induced cell differentiation according to different markers.
Kang et al., 2011 [46]	L-PRF	Human alveolar bone marrow stem cells (hABMSCs)	0, 0.5, 3, 6, and 12 h. 1, 7, 14, 21, 28 and 35 days	Eluate	Proliferation; mineralization; migration; MMP9 activity.	PRF increased the proliferation, aggregation, activation of MMP9, and mineralization by decreasing the migration of the hABMSCs.
Kardos et al., 2018 [47]	Fresh, frozen, and freeze-dried L-PRF	Mesenchymal stem cells	1, 7 and 14 days	Co-culture	Viability; proliferation; adhesion.	Preserved PRF membranes presented the same biological properties as fresh samples.
Kim et al., 2017 [48]	L-PRF	Human primary osteoblasts	1, 2, 3 and 7 days	Co-culture	Proliferation; ALP activity.	PRF presented significantly higher data on DNA quantification, synthesis and proliferation, differentiation, and bone generation of osteoblasts, PDGFs, and TGF-b.
Kim et al., 2017 [49]	L-PRF	Human dental pulp cells (HDPCs)	1, 2 and 3 days	Eluate	Viability; IL-1b; IL-6, and IL-8; VCAM-1, DSP; DMP-1; ALP activity; mineralization.	PRF presents odontogenic capacity in inflamed HDPCs.
Koyanagi et al., 2022 [50]	Arterial blood-derived PRF (Ar-PRF) and venous blood-derived PRF (Ve-PRF)	Primary rabbit osteoblasts	1, 3 and 5 days	Eluate	Viability; Col-1; OCN; mineralization.	Exposed osteoblasts presented greater differentiation potential, including higher osteocalcin expression and mineralization with no difference between Ar and Ve-PRF.
Kosmidis et al., 2023 [51]	A-PRF, i-PRF, and L-PRF	Human osteosarcoma osteoblast-like cells (U2OS)	Up to 28 days	Eluate	Mineralization; ALP activity; ALP; OCN; ON; ICAM-1; Runx2; Col 1a.	The three PRF preparations increased the osteogenic potential of U2OS cells. A-PRF presented the highest effect on mineralization, and i-PRF had the highest potential for early cell differentiation.
Kyyak et al., 2020 [52]	i-PRF	Human osteoblasts (HOBs)	3, 7 and 10 days	Eluate	Viability; migration; proliferation; ALP; BMP-2; OCN.	i-PRF in combination with allogenic biomaterials enhances human osteoblast activity compared to xenogenic bone substitute material + i-PRF.
Kyyak et al., 2021 [53]	i-PRF	Human osteoblasts (HOBs)	3, 7 and 10 days	Eluate	Viability; migration; proliferation; ALP; BMP-2; OCN.	The combination of four bovine bone substitute materials with i-PRF improved all cellular parameters, ALP and BMP-2 expression at earlier stages, and osteonectin expression at later stages.
Li et al., 2013 [54]	L-PRF	Dental follicle (DF), alveolar bone (AB), and periodontal ligament (PDL)	7, 14 and 21 days	Co-culture	Proliferation; migration; mineralization; ALP; MGP; Runx2.	PRF induced an increase in the early osteoblast transcription factor Runx2 and a reduction in the mineralization inhibitor MGP.
Li et al., 2014 [55]	Lyophilized PRF	Rat alveolar bone cells	7, 14 and 21 days	Co-culture	Mineralization; proliferation; ALP; Runx2.	Lyophilized PRF caused greater proliferation and elevation in the Runx2 expression in alveolar bone cells compared to fresh PRF and a more than 10-fold rise of ALP and in vitro mineralization.
Li et al., 2018 [56]	L-PRF	Human periodontal ligament stem cells (hPDLCs)	21 days	Exudates	Adhesion; proliferation; mineralization; ALP activity; ALP; OCN; OSX; Runx2.	PRF exudate enhances hPDLC adhesion and proliferation and induces the differentiation of hPDLCs into mineralized tissue formation cells.
Li et al., 2018 [57]	L-PRF	Human periodontal ligament cells (PDLSCs)	1, 2, 3, 7 and 14 days	Co-culture	Proliferation; Runx2; MAPK; ERK1/2; pERK1/2; JNK1/2/3; pJNK1/2/3; P38; OSX; OCN; ALP activity.	PRF and IGF-1 can promote the osteogenic differentiation of PDLSCs and enhance their osteogenic mineralization through the regulation of the MAPK pathway.
Liu et al., 2022 [59]	Lyophilized L-PRF, crosslinked with genipin	Pulp stem cells from human exfoliated deciduous teeth (SHEDs)	Up to 14 days	Eluate	Proliferation; mineralization; Runx2; Col 1; OCN.	Genipin crosslinked L-PRF induced cell proliferation and enhanced the expression of key genes in osteogenesis.
Liang et al., 2021 [58]	A-PRFe	Adipose-derived stem cells (ASCs)	7 days	Eluate	Roliferation; mineralization; adipogenesis; ALP, OPN; OCN; Runx2.	A-PRF stimulated ASC proliferation and adipogenic and osteogenic differentiation in a dose-dependent manner.
Liu et al., 2019 [60]	Fresh/lyophilized PRF	Bone mesenchymal stem cells (BMSCs)	1–7 days	Eluate	Proliferation; mineralization.	Fresh/lyophilized PRF (1:1) increased BMSC proliferation and in vitro mineralization.
Lo Monaco et al., 2020 [61]	L-PRF	Dental pulp stem cells (DPSCs)	24, 48 and 72 h	Eluate	Chondrogenic differentiation; TIMP-1; proliferation.	L-PRF induced differential chondrogenesis on DPSCs.
Marchetti et al., 2020 [62]	L-PRF	Periodontal ligament fibroblasts	24 h, 72 h and 7 days	Eluate	Proliferation; viability; morphology.	L-PRF stimulated the onset of the growth of the periodontal ligament fibroblasts.
Moradian et al., 2017 [63]	L-PRF	Bone marrow mesenchymal stem cells (BMMSCs)	1, 5, 7, 9 and 12 days	Cultured on PRF	Proliferation; adhesion.	PRF significantly induced BMMSC proliferation. Scanning electron microscopy showed that BMMSCs tightly adhered to the fibrin scaffold after seeding.
Nguyen et al., 2022 [64]	A-PRF	Human periodontal ligament stem cells (hPDLSCs)	1 h, 6 h, 24 h, 3 days, 5 days and 7 days	Exudates	Proliferation; migration.	A-PRF in combination with xenogenic bone induced hPDLSC migration or proliferation, depending on the exudate concentration.
Nie et al., 2020 [65]	Lyophilized L-PRF	MC3T3-E1 murine preosteoblasts	1, 3 and 5 days	Eluate	Proliferation; mineralization; OCN; OPN.	Eluates from lyophilized PRF added as a component for electrospinning preparation enhanced the proliferation, mineralization, and expression of the OCN and OPN of MEC3T3-E1 cells.
Nugraha et al., 2018a [66]	L-PRF	Rat gingival mesenchymal stem cells (GMSCs)	7, 14 and 21 days	Cultured on PRF	ALP; OC.	PRF induced increased ALP and OCN expression on GMSCs.
Nugraha et al., 2018b [67]	L-PRF	Rat gingival somatic cells (GSCs)	7, 14 and 21 days	Cultured on PRF	BSP-1.	PRF increases and stimulates GSC BSP-1 expression.
Nugraha et al., 2018c [68]	L-PRF	Rat gingival stromal progenitor cells (GSPCs)	7, 14 and 21 days	Cultured on PRF	Cbfa-1; sox9.	GSPCs cultured in PRF possessed a potential osteogenic differentiation ability, as predicted by the cbfa-1/sox9 expression ratio.
Nugraha et al., 2019 [69]	L-PRF	Rat gingival mesenchymal stem cells (GMSCs)	7, 14 and 21 days	Cultured on PRF	Aggrecan.	Platelet-rich fibrin increases the aggrecan expression of GMSCs during osteogenic differentiation.
Rastegar et al., 2021 [70]	L-PRF	Human osteosarcoma osteoblast-like cells (MG-63)	3 days	Co-culture	ALP activity; mineralization.	PRF loaded into PCL/chitosan core-shell fibers promoted in vitro mineralization and increased ALP activity.
Shah et al., 2021 [71]	i-PRF	Human osteosarcoma osteoblast-like cells (MG-63)	1, 7, 14 and 21 days	Co-culture	Proliferation; ALP activity; mineralization.	Coating titanium discs with i-PRF causes increased proliferation, alkaline phosphatase production, and mineralization at days 1, 7, 14, and 21.
Song et al., 2018 [72]	L-PRF	Rabbit bone marrow-derived mesenchymal stem cells (BMSCs)	7 days	Eluate	Adhesion; proliferation; ALP; Col-1; OPN; Runx2.	Printed scaffolds of BCP/PVA associated with PRF promoted the adhesion, proliferation, and differentiation of BMSCs.
Steller et al., 2019 [73]	L-PRF	Human osteoblasts (HOBs)	72 h	Eluate	Proliferation; migration; viability.	The use of PRF improves the behavior of osteoblasts treated with zoledronic acid.
Steller et al., 2019 [74]	PRF	Human primary osteoblasts	24 h	Eluate	Adhesion; viability; morphology.	Zoledronic acid decreased osteoblast adhesion on implant surfaces. PRF increased the primary adhesion of zoledronic acid-treated osteoblasts on implant surfaces in vitro.
Sui et al., 2023 [75]	3D-printed L-PRF composite scaffolds	MC3T3-E1 murine preosteoblasts	1 to 3 days	Cultured on a PRF composite scaffold	Proliferation.	The proliferation of preosteoblasts into the scaffolds increased with the release of GFs, indicating that L-PRF remains bioactive after 3D printing.
Thanasrisuebwong et al., 2020 [76]	Subfractioned (red and yellow) i-PRF	Periodontal ligament stem cells	0, 3 and 5 days	Eluate	Proliferation; migration; mineralization; ALP activity.	The factors released from the red i-PRF had a greater effect on cell proliferation and cell migration, while yellow i-PRF stimulated earlier osteogenic differentiation of periodontal ligament stem cells.
Verboket et al., 2019 [77]	High (208 g) and low (60 g) RCF PRF	Bone marrow mononuclear cells (BMCs)	2, 7 and 14 days	Eluate	Viability; apoptosis; VEGFA; ICAM3; MMP2; MMP7; MMP9; TGF-β1; BCL2; BAX; ALP; COL-1; FGF2; SPP1.	PRF produced with low RCF significantly increased mediator contents and stimulatory effects on BMC with regard to the gene expression of MMPs and metabolic activity/viability.
Wang et al., 2015 [78]	L-PRF	Rabbit mesenchymal stem cells (MSCs)	1, 2, 3, 4, 5, 6, 7, 8 and 14 days	Eluate	Proliferation; ALP; BMP2; OCN; OPN; Col-1.	PRF significantly stimulated MSC proliferation and osteogenesis in vitro.
Wang et al., 2018 [79]	i-PRF	Human primary osteoblasts	1, 3, 5, 7 and 14 days	Co-culture	Proliferation; migration; adhesion; mineralization; ALP activity; ALP; Col-1; OC.	i-PRF was able to influence osteoblast behavior, including migration, proliferation, and differentiation, at higher levels than PRP.
Wang et al., 2022 [80]	i-PRF	Human bone marrow stem cells (hBMSCs)	1 to 7 days	Eluate	Proliferation; survival; migration; mineralization; Col 1; OCN; OPN; Runx2; ERK 1/2; p-ERK.	i-PRF improved the proliferation and migration of hBMSCs, with an increased expression of osteogenic markers, mineralization, and activation of the ERK pathway.
Wang et al. 2022 [81]	L-PRF	Rabbit mesenchymal stem cells from the Schneiderian membrane (SM-MSCs)	1 to 14 days	Eluate	Proliferation; migration; mineralization; ALP activity; ALP; Col 1; Runx2; ERK 1/2; p-ERK.	PRF stimulated proliferation, migration, and osteogenic differentiation of SM-MSCs, with the upregulation of the ERK 1/2 signaling pathway.
Wong et al., 2021 [7]	Large-pore PRF (LPPRF)	MC3T3-E1 preosteoblasts	6 days	Eluate	Proliferation; migration; mineralization.	Large-pore LPPRF combined with a Mg ring increased preoteoblast proliferation, migration, and in vitro calcium deposition.
Wong et al., 2021 [82]	L-PRF	Rabbit primary osteoblasts	3 and 6 days	Eluate	Viability; ALP activity; Col-1; OPN; ALP.	L-PRF positively affected primary osteoblast behavior and induced bone formation when associated with TCP.
Woo et al., 2016 [83]	L-PRF	Human dental pulp cells (HDPCs)	12 h. 1, 2 and 7 days	Eluate	Viability; ALP activity; DSP; DMP1; BMP 2/4; pSmad1/5/8.	A combination of MTA and PRF synergistically stimulated odontoblastic differentiation of HDPCs by the modulation of the BMP/Smad pathway.
Wu et al., 2012 [84]	L-PRF	Human osteosarcoma osteoblast-like cells (U2OS)	2 h. 1, 3 and 5 days	Co-culture	Adhesion; proliferation p-Akt; HSP47; LOX.	PRF increased cell attachment and proliferation by the Akt pathway and matrix synthesis via HSP47 and LOX accumulation.
Yu et al., 2016 [85]	L-PRF	Canine deciduous and permanent dental pulp cells (DPCs)	1, 4, 7 and 11 days	Co-culture	Cytotoxicity; proliferation; ALP activity; mineralization; Col-1; OCN; OPN; Runx2, ALP.	PRF stimulated the proliferation and differentiation of both deciduous and permanent DPCs, and deciduous pulp cells were more responsive to the effects of PRF.
Yu et al., 2023 [86]	H-PRF	Human osteoblasts (hFOBs)	3 days	Transwell inserts	Migration.	The culture medium from H-PRF bone blocks markedly promoted the migration of osteoblasts.
Zhang et al., 2019 [87]	L-PRF	Dental pulp stem cells (DPSCs)	1, 3, 5 and 7 days	Eluate	Migration; morphology; ALP activity; mineralization (SEM); OPN; Col-1; ALP.	Multifunctional triple-layered scaffolds combined with PRF significantly increased ALP activity and the expression of differentiation markers on DPSCs.
Zhang et al., 2023 [88]	i-PRF	Human dental pulp stem cells (hDPSCs)	Up to 21 days	Eluate	Proliferation; mineralization; ALP activity; Runx2; DSPP; DMP1; BSP; Notch 1; Jagged 1; Hes 1.	I-PRF induced a dose-dependent increase in the proliferation of hDPSCs and the expression of osteo-/odontoblastic differentiation markers, as well as key proteins in the Notch signaling.
Zhao et al., 2013 [89]	L-PRF	Periodontal ligament stem cells (PDLSCs)	7, 14 and 21 days	Co-culture	Proliferation; mineralization (SEM); ALP activity; BSP; OCN; Col-I; CP23.	PRF induced proliferation in PDLSCs while suppressing the osteoblastic differentiation of PDLSCs by decreasing ALP activity and the gene expression of BSP and OCN while upregulating the mRNA expression levels of Col-I and CP23 during the testing period.
Zheng et al., 2015 [90]	Lyophilized PRF	Human osteosarcoma osteoblast-like cells (MG63)	1, 3 and 5 days	Co-culture	Viability; adhesion; proliferation.	A combination of hydrogel and a nanostructured scaffold loaded with PRF improved the adhesion and proliferation of MG63 cells compared to the controls.
Zheng et al., 2020 [91]	i-PRF	Human periodontal ligament cells (hPDLCs)	1, 3 and 5 days	Eluate	Migration; proliferation; ALP activity; mineralization; Runx2; Col-1; OCN; IL-1β; TNF-α and p65 (in the presence of LPS).	Liquid PRF promoted hPDLÇ proliferation and differentiation and attenuated the inflammatory state induced by LPS.

**Table 4:** The main data extracted from the studies with PRF associated with other materials and/or compounds.

References	Associated Material	Relevance	Results
Al-Maawi, 2021 [17]	OsteoporeTM (OP), a commercially available PCL mesh	Combination of differently centrifuged PRF matrices with a polymeric resorbable scaffold to influence their biological properties on bone regeneration.	The presented results suggest that PRF produced according to the low-speed centrifugation concept exhibits autologous blood cells and growth factors and seems to have a significant effect on osteogenesis, showing promising results to support bone regeneration.
Chi, 2019 [26]	Chitosan/gelatin (C/G)	Test whether decellularized PRF (DPRF) maintains its bioactive effects to improve chitosan/gelatin (C/G) base scaffolds, which display appropriate biocompatibility and mechanical properties but lack biological activity to promote soft and hard tissue repair.	C/G/DPRF scaffolds accelerated attachment, proliferation, and osteogenesis-related marker expression of bone marrow stem cells. In vivo, C/G/DPRF scaffolds led to enhanced bone healing and defect closure in a rat calvarial defect model. Thus, it was concluded that DPRF remains bioactive, and the prepared C/G/DPRF scaffold is a promising material for bone regeneration.
Douglas, 2012 [30]; Gassling, 2013 [37]	Calcium glycerophosphate (CaGP) and ALP	Induce the mineralization of PRF membranes to achieve mechanical reinforcement of the gel and stability as a barrier membrane in guided bone regeneration.	The mineralization was confirmed, and WST test results showed that cell proliferation was inferior on PRF after the addition of ALP, confirming its properties as a barrier.
Girija and Kavitha, 2020 [39]	Bioactive radiopacifiers—nanohydroxyapatite (nHA) and dentin chips (DCs)	Combine bioactive radiopacifiers, nanohydroxyapatite (nHA) and dentin chips (DC), to PRF, aiming to produce a traceable material for endodontic procedures while still inducing adequate biological responses.	The results suggest that the addition of bioactive radiopacifiers into PRF has a synergistic effect on the stimulation of odontoblastic differentiation of HDPCs, inducing mineralization.
Ji B, 2015 [45]	Treated dentin matrix (TDM)	Associate endogenous stem cells, PRF, and TDM in the local microenvironment to contribute to the regeneration of periodontal tissues around the tooth root.	The study confirmed the role of PRF as a bioactive agent with TDM as an inductive scaffold for cells of the tooth socket microenvironment involved in endogenous tooth root regeneration.
Kyyak, 2020 [52]	Allogenic (ABSM) and xenogenic bone substitute material (XBSM)	The comparison of allogenic and xenogenic bone substitutes with i-PRF for the production of the more bioactive composite material for bone treatment.	i-PRF in combination with ABSM enhances HOB activity compared to XBSM-i-PRF or untreated BSM in vitro. Therefore, the addition of i-PRF to ABSM and—to a lower extent—XBSM may influence osteoblast activity in vivo in an interesting way for bone therapy.
Kyyak, 2021 [52]	Cerabone R (CB), Bio-Oss R (BO), Creos Xenogain R (CX), and MinerOSS R X (MO)	Four bovine bone substitute materials (XBSMs) were associated with i-PRF and aimed to increase their osteoinductive properties.	XBSM sintered under high temperatures showed increased HOB viability and metabolic activity throughout the whole period compared to XBSM manufactured at lower temperatures. Overall, the combination of XBSM with i-PRF improved all cellular parameters related to osteogenesis.
Nguyen, 2022 [64]	Xenogenic bone substitute material (XBSM)	Advanced platelet-rich fibrin (A-PRF) and xenogenic bone substitute material (XBSM) were associated and aimed to increase periodontal tissue regeneration.	The PRF-XBSM mixture continuously released growth factors over 7 days and enhanced human ligament stem cell proliferation and migration.
Nie, 2020 [65]	Polyvinyl alcohol/sodium alginate	The addition of lyophilized PRF as a component for electrospinning preparations to increase the proliferation and osteogenesis of osteogenic precursor cells for bioengineering purposes.	The resulting material presented adequate physicochemical properties and was able to increase osteogenic markers on bone cells.
Rastegar, 2021 [70]	PCL/chitosan	Platelet-rich fibrin (PRF)-loaded PCL/chitosan (PCL/CS-PRF) core-shell nanofibrous scaffold was made through a coaxial electrospinning method. The goal was to evaluate the effect of CS-RPF in the core layer of the nanofibrous on the osteogenic differentiation of human mesenchymal stem cells (HMSCs).	The formation of Ca-P on the surface of the scaffold immersed in a simulated body fluid solution indicated the suitable osteoconductivity of the PCL/CS-PRF core-shell nanofibrous scaffold. Due to the higher hydrophilicity and porosity of the PCL/CS-PRF core-shell nanofibrous scaffold compared to the PCL/CS scaffold, better bone cell growth on the surface of the PCL/CS-PRF scaffold was observed.
Song, 2018 [72]	Nano-biphasic calcium phosphate (BCP) and polyvinyl alcohol (PVA)	The low-temperature 3D printing of BCP/ PVA/PRF scaffolds would preserve the biological activity of PRF and provide an innovative biomaterial for restoring segmental bone defects.	The biological activity of PRF was retained during the 3D printing process, and the presence of PRF in the biocompatible microenvironment of the scaffold provided cell binding sites and promoted the adhesion, proliferation, and the differentiation of BMSCs.
Steller, 2019 [73]	Zoledronic acid	An investigation of the effects or bone cells treated with bisphosphonates as a potential mitigator of osteonecrosis associated with treatments with these drugs.	The negative effects of ZA on osteoblast survival and behavior (proliferation, morphology, adhesion to implant surface) were especially reduced using PRF, indicating that the autologous material may have positive effects in the therapy of bisphosphonate-related osteonecrosis of the kaw.
Sui et al., 2023 [75]	Chitosan (CS)–hydroxyapatite (HAP) scaffolds	A study aiming to identify if the 3D printing of a CS-HAP-PRF would compromise the biological properties of the platelet aggregate.	Based on the presented experimental results, it is possible to infer that the 2.5% P-C-H scaffold exhibits remarkable biological activity. And, therefore, it is not negatively affected by 3D printing.
Woo, 2016 [83]	Mineral trioxide aggregate (MTA)	Combined PRF as a bioactive matrix and MTA as a root-filling material beneficial for the endodontic management of an open apex.	The combination of MTA and PRF was proven as an odontogenic inducer in human dental pulp cells (HDPCs) in vitro.
Wong, 2021 [7]	Magnesium rings	The freeze-drying enlarges the pores of PRF to engineer large-pore PRF (LPPRF), a type of PRF that has expanded pores for cell migration. Biodegradable Mg rings were used to provide stability to these pores and release Mg ions during degradation, with the potential to enhance osteoconduction and osteoinduction.	The results revealed that cell migration was more extensive when LPPRF was used rather than PRF. Moreover, the Mg ions released from the Mg rings significantly enhanced the calcium deposition by preosteoblasts, evidencing in vitro osteoinduction.
Wong, 2021 [82]	Tricalcium phosphate (TCP)	The development of a composite biomaterial combining the osteoconductive TCP incorporated with bioactive PRF for bio-synergistic bone regeneration.	The in vitro results showed that PRF plus TCP had excellent biosafety and was favorable for increasing osteoblast activity related to bone repair.
Zhang, 2019 [87]	Polycaprolactone, chitosan, and hydroxyapatite	Polycaprolactone/gelatin (PG) nanofiber films by electrospinning chitosan/poly (γ-glutamic acid)/hydroxyapatite (CPH) hydrogels were formed by electrostatic interaction and lyophilization to exert osteoconduction, and platelet-rich fibrin (PRF) was added to promote bone induction through the release of growth factors.	This study provided evidence that the composite biomaterial positively affects dental pulp stem cells, with great potential for endodontics and wider applications, such as calvarial repair and oral alveolar bone regeneration.
Zheng, 2015 [90]	Copolymer poly-polyethylene glycol (PEG)-PLGA (PLGA-PEG-PLGA)	A combination of PRF with PLGA and nano-hydroxyapatite (nHA/PLGA) might produce a scaffold with high porosity, controlled pore size to better mimic natural bone, and improved osteogenic ability.	The resulting scaffold provided a good substrate for osteoblast proliferation with sustained-release growth factors, producing a promising therapeutic agent for local applications in bone tissue engineering.

## Data Availability

No new data were created.
